# Where experience makes a difference: teachers’ judgment accuracy and diagnostic reasoning regarding student learning characteristics

**DOI:** 10.3389/fpsyg.2024.1278472

**Published:** 2024-03-07

**Authors:** Christian Kosel, Elisabeth Bauer, Tina Seidel

**Affiliations:** Friedl-Schöller Endowed Chair for Educational Psychology, TUM School of Social Sciences and Technologies, Technical University of Munich (TUM), Munich, Germany

**Keywords:** judgment accuracy, professional vision, student characteristics, expert-novice comparison, noticing, reasoning

## Abstract

The concept of teacher professional vision suggests that experienced teachers, compared to novice teachers, might be better at making accurate judgments of students’ learning characteristics, which can be explained by their advanced reasoning in diagnostic situations. This study examines experienced and novice teachers’ diagnoses of different student characteristic profiles: three inconsistent profiles (overestimating, uninterested, and underestimating) and two consistent profiles (strong and struggling). We examined both experienced (*n* = 19 in-service mathematics teachers) and novice teachers (*n* = 24 pre-service mathematics teachers) to determine the extent of differences in their judgment accuracy and their diagnostic reasoning about observable cues when diagnosing student profiles while watching a lesson video. ANOVA results indicate that experienced teachers generally achieved a higher judgment accuracy in diagnosing student profiles compared to novice teachers. Moreover, epistemic network analysis of observable cues in experienced and novice teachers’ diagnostic reasoning showed that, compared to novice teachers, experienced teachers make more relations between a broader spectrum of both surface cues (e.g., a student’s hand-raising behavior) and deep cues (e.g., a student being interested in the subject). Experienced teachers thereby construct more comprehensive and robust reasoning compared to novice teachers. The findings highlight how professional experience shapes teachers’ professional skills, such as diagnosing, and suggest strategies for enhancing teacher training.

## 1 Introduction

In day-to-day teaching, teachers constantly gather real-time information about their students that enables them to provide personalized instruction. Based on their observations of student learning behavior, they adjust the difficulty of ongoing learning tasks, provide feedback, and assess student performance (Corno, 2008). Judging the cognitive and motivational-affective learning characteristics of students has been identified as a fundamental aspect of teachers’ daily professional work: As stated by Shavelson (1978, p. 37), “teachers’ estimates of students ‘states of mind’—cognitive, emotional, motivational—provide primary information in deciding how to teach” and “during teaching itself, new information can be obtained bearing on the student’s current state of mind.” In this context, several educational researchers have endeavored to address the question of how accurately teachers can judge student characteristics that are relevant to learning. Meta-analyses by Machts et al. (2016) and Südkamp et al. (2012) found that teachers exhibit relatively high accuracy in judging students’ cognitive abilities and learning achievements. However, when it comes to motivational-affective characteristics, such as self-concept and interest, challenges arise, and teachers’ accuracy tends to fluctuate (Spinath, 2005). Nevertheless, previous studies have predominantly focused on evaluating the accuracy of teachers in judging single—isolated—student characteristics, potentially overlooking the holistic nature of how teachers perceive students and make judgments by considering multiple learning-relevant characteristics (Kaiser et al., 2013).

To overcome this limitation, a novel line of research has emerged, focusing on exploring the accuracy of teachers in judging complex student profiles (Huber and Seidel, 2018; Südkamp et al., 2018; Schnitzler et al., 2020). Recognizing the interconnectedness of various student characteristics in a latent student profile, this new approach seeks to understand how teachers can effectively integrate and evaluate a range of characteristics simultaneously. Regarding teachers’ judgment accuracy, preliminary evidence suggests that teachers tend to overestimate the consistency of student profiles and face challenges in identifying student profiles with conflicting information about cognitive and motivational-affective characteristics (e.g., a student with high cognitive ability but low self-concept; Huber and Seidel, 2018; Südkamp et al., 2018). To explain variations in teacher judgment accuracy when diagnosing student profiles, research has focused on teacher professionalization and expertise, indicated by teachers’ professional experience (Seidel et al., 2020). To understand how experience can affect judgment in diagnosing student profiles, it is essential to delve into teachers’ diagnostic reasoning to gain insight into the role of experience and its potential influence on the judgment accuracy of student profiles.

The present study builds on previous studies (Huber and Seidel, 2018; Südkamp et al., 2018; Schnitzler et al., 2020; Seidel et al., 2020) by employing an expert-novice paradigm to examine teachers’ judgment accuracy in the context of diagnosing student engagement and underlying latent student profiles. The study focuses on five distinct student profiles, including three inconsistent profiles (overestimating, uninterested, and underestimating) and two consistent profiles (strong and struggling). By delving into teachers’ reasoning as grounded in the framework of teacher professional vision (Seidel and Stürmer, 2014), this study establishes a new perspective on the differences between experienced and novice teachers when diagnosing student profiles.

### 1.1 Teachers’ diagnosing of student learning characteristics

In the educational context, teachers’ *diagnosing* is characterized by teachers’ assessment of their students’ diverse characteristics and learning needs (Artelt and Gräsel, 2009). Research focusing on teachers’ diagnosing is mainly interested in three kinds of teacher *judgment accuracy*, which refers to their performance in accurately judging student characteristics: Firstly, research about teachers’ task-related judgment accuracy, focuses on teachers’ ability to judge the difficulty of tasks based on the collective performance of the class (McElvany et al., 2009). Secondly, research about teachers’ person-specific judgment accuracy delves into teachers’ ability to judge individual student behaviors, including mental disorders (Mathews et al., 2020) and learning difficulties in mathematics (Kilday et al., 2011). Thirdly, especially in recent years, there has been increasing research interest in teachers’ person-related judgment accuracy, which includes teachers’ judgment of various cognitive and motivational-affective characteristics of students. These include but are not limited to, subject-specific self-concept (Helm et al., 2018), achievement (Südkamp et al., 2012), and cognitive ability (Machts et al., 2016). On the one hand, research highlights the positive correlations between student characteristics, learning behaviors, and academic achievements (Wigfield and Eccles, 2000). On the other hand, research emphasizes how teachers’ tailored instructional methods (e.g., level of support, feedback, or task choice) can positively influence these student characteristics (Schrader and Helmke, 1987; Corno, 2008; Urhahne and Wijnia, 2021). However, to optimally support student development, teachers need to accurately assess student characteristics (Urhahne and Wijnia, 2021). Research has shown that teachers vary in their accuracy in judging various student characteristics. They are generally more accurate at judging cognitive student characteristics in terms of academic achievement, with correlations between teacher judgments and actual student achievement ranging between *r* = 0.20 and *r* = 0.90 (median *r* = 0.66; Hoge and Coladarci, 1989; Machts et al., 2016). On the other hand, judging motivational-affective student characteristics, such as test anxiety and academic self-concept, are less accurate, with correlations ranging from *r* = −0.39 to *r* = 0.82 (median *r* = 0.39; Spinath, 2005). When considering this statistical synthesis of study results, it is important to note that the studies included in meta-analytic approaches vary widely in terms of methodological study characteristics (direct vs. indirect ratings, norm-referenced vs. peer-dependent ratings, measures of constructs). However, as Urhahne and Wijnia (2021) summarized in their synthesis of 40 years of research on teacher judgment, teacher judgments in areas other than student achievement often have relatively low levels of accuracy. Moreover, there is a significant gap in these studies as they primarily focus on single student characteristics in isolation and therefore, neglect the interconnected nature of student characteristics within students.

### 1.2 Diagnosing student profiles

Evidence suggests that teachers often perceive students holistically, interweaving different student characteristics when asked to judge specific aspects, such as student achievement or motivation (Südkamp et al., 2018). For example, Kaiser et al. (2013) observed that teachers’ judgments are not limited to individual student characteristics, but are influenced by their perceptions of other student characteristics as well. Using structural equation modeling, the authors showed that teachers used students’ achievement to make judgments about students’ level of motivation. Evidence from variable-centered analyses shows that student characteristics are indeed significantly correlated (e.g., levels of academic achievement and prior knowledge; Schrader and Helmke, 2008). However, judging one characteristic based on another characteristic can result in a biased judgment (also referred to as halo effect; Fiedler et al., 2002), for example, when a student’s cognitive and motivational-affective characteristics are not consistently high or low.

To explore the dynamics of student characteristics within individuals while simultaneously mapping the diversity of student characteristics among different groups, person-centered analyses gained increasing attention (Seidel, 2006; Linnenbrink-Garcia et al., 2012; Kosel et al., 2020). Person-centered analyses go beyond examining isolated student characteristics (i.e., variable-centered analyses) by integrating different student characteristics and describing their inherent structure within a person, such as a student (Lubke and Muthén, 2005). In education, person-centered approaches are used to identify different *student profiles* based on their unique combination of student characteristics. Research has uncovered a variety of student profiles with varying combinations of cognitive and motivational-affective student characteristics (Seidel, 2006; Linnenbrink-Garcia et al., 2012; Kosel et al., 2020). For instance, Kosel et al. (2020) analyzed data on about 10.000 German 9th-grade students’ cognitive abilities, prior knowledge, self-concept, and interest in the two subjects of mathematics and language arts. Using latent profile analysis, they showed for both subjects that some groups of students have *consistent profiles* of cognitive and motivational-affective characteristics and can be categorized as either “strong students” or “struggling students,” meaning they have consistently high or low values in student characteristics data. Other students exhibit *inconsistent profiles*, such as “underestimating students” (knowledgeable but lacking confidence in their self-concept of ability), “overestimating students” (less knowledgeable but highly confident in their self-concept of ability), or “uninterested students” (overall knowledgeable and confident but with limited interest in a subject area). Regarding the distribution of profiles, the underestimating student profile was prevailing in both subjects (around 35 percent of the students), with the second highest prevalence being observed for the struggling profile in mathematics (around 24 percent) and the overestimating profile in language arts (around 27 percent). The findings thus indicate that teachers in varying subjects are often confronted with students having inconsistent profiles of cognitive and motivational-affective characteristics.

Huber and Seidel (2018) as well as Südkamp et al. (2018) explored student profiles by comparing teacher and student perceptions regarding the interplay of cognitive and motivational-affective student characteristics. In both studies, the authors found that teachers’ perceptions were dominated by homogeneous sets of average student characteristics. For example, Südkamp et al. (2018) found that teachers tend to rate their students consistently as either above average, average, or below average on cognitive and motivational-affective student characteristics; in contrast, students’ ratings indicated a diverse and sometimes inconsistent interplay of student characteristics. Thus, teachers seem to struggle with decoupling different student characteristics but instead tend to assume consistency in student profiles—although, contrary to the authors’ expectations, teachers’ judgments were not more accurate for consistent compared to inconsistent student profiles.

### 1.3 The role of professional experience

The tendency toward assuming consistency between student characteristics can be ascribed to teachers’ cognitive processes. Südkamp et al. (2018) acknowledge the role of heuristic information processing—an automatic, unconscious, and, thus, efficient processing of information (see Evans, 2008)—in teacher judgments. Heuristic information processing is generally favored under situational conditions, such as limited time or information to act on (Chaiken and Trope, 1999), which are common conditions of teaching situations. *Heuristics* are mental shortcuts that simplify cognitive inferences (Kahneman and Klein, 2009; Norman et al., 2017). They can result in biased judgment (e.g., halo effect; Fiedler et al., 2002) but can also be highly functional if based on professional knowledge and experience (e.g., Boshuizen et al., 2020).

*Professional knowledge* initially consists of the knowledge that novice teachers learn in the course of their studies, which is later elaborated and restructured into higher-order representations through professional experience (Boshuizen et al., 2020). One such knowledge representation is cognitive prototypes, which are representations of categories (e.g., “students”) with typical attributes (e.g., student characteristics) or patterns of attributes (e.g., student profiles) that were abstracted from experience (Cantor and Mischel, 1977; Hörstermann et al., 2010; Papa, 2016). With increasing experience, teachers are exposed to a greater number and a greater variety of students, allowing them to refine their cognitive prototypes of typical student characteristics and student profiles (Boshuizen et al., 2020). Drawing on their elaborated professional knowledge, experienced teachers thus have superior prerequisites for accurately diagnosing student profiles. This assumption was supported in a study by Seidel et al. (2020), in which teachers were asked to assign students to consistent and inconsistent student profiles based on an authentic video vignette about the students’ learning behavior. The results indicated a higher accuracy on the side of experienced teachers compared to novice teachers in judging student profiles. However, other existing studies report heterogeneous results regarding the influence of professional experience on judgment accuracy (Royal-Dawson and Baird, 2009; Ready and Wright, 2011); for instance, Ready and Wright (2011) asked teachers with different levels of experience to predict students’ test scores and found lower correlations between predicted and actual scores for more experienced teachers. These studies emphasize that teachers’ experience does not necessarily lead to higher judgment accuracy but other factors, for example, relating to diagnostic processes, are relevant to consider as well.

Some studies investigated cognitive processing in teachers’ diagnosing. These studies have shown that experienced teachers with elaborated professional knowledge are better able to constantly monitor the responses and activities of all students in class, while at the same time being alert to those students and events that might require particular actions or adaptations during teaching (Clarridge and Berliner, 1991; Stahnke and Blömeke, 2021; Wolff et al., 2021; Kosel et al., 2023). Goodwin (1994) characterized this phenomenon as *professional vision*—a concept that was further elaborated by researchers, such as van Es and Sherin (2010) and Seidel and Stürmer (2014). Professional vision denotes the ability of teachers to effectively engage in cognitive and behavioral facets of classroom observation, which shapes their instructional practices and decision-making in educational contexts. Seidel and Stürmer (2014) distinguished two fundamental dimensions of professional vision: *noticing* student behavior by directing attention to relevant information; and *reasoning*, which is the cognitive interpretation of the collected information. Experienced teachers’ elaborated knowledge drives their ability to notice relevant cues or factors that novices may miss (Clarridge and Berliner, 1991). Moreover, elaborated professional knowledge facilitates teachers’ reasoning in terms of seamlessly integrating situational information with their professional knowledge (Wolff et al., 2021), which can lead to more nuanced and accurate judgments than novice teachers who had limited exposure to the intricacies of the profession. Thus, when diagnosing student profiles, teachers’ reasoning underlying their final judgment is influenced by their professional knowledge and experience regarding student characteristics and typically occurring student profiles.

### 1.4 Teachers’ diagnostic reasoning

Student learning characteristics and their integration into student profiles are not directly observable but represent latent constructs, which teachers diagnose through reasoning about noticed cues regarding the students’ behavior (Back and Nestler, 2016). To underscore the crucial role of observable cues in shaping teachers’ judgments, recent models of teacher judgment (Herppich et al., 2018; Loibl et al., 2020) referred to the lens model proposed by Brunswik (1955). As teachers observe and interpret a myriad of observable cues, they construct mental representations of students’ latent characteristics as a basis for making informed judgments. For example, in a diagnostic situation where a teacher is judging a student’s self-concept, the teacher identifies observable cues—such as behaviors (e.g., lack of eye contact) and interactions (e.g., avoidance of group activities)—that may indicate the student’s self-concept. The teacher correlates these various cues as an indicator of the student’s self-concept, thereby validating the cues with each other and making a probabilistic yet informed judgment about the student’s self-concept as a latent construct. Some cues can be characterized as *surface cues* (Brunswik, 1955; Loibl et al., 2020) because they are directly observable. As indicated by prior research on classroom management, such surface cues—for example, overt signs of disinterest (e.g., playing with a pen) or disruptive behavior (e.g., talking to other students or throwing around things)—are easily perceived by teachers (Stürmer et al., 2017). In contrast, *deep cues* require making some interpretation from direct observations. For example, remaining quiet in the classroom can be an indicator of low self-concept but also low motivation (Seidel et al., 2016). Such deep cues are often challenging for teachers to evaluate (Kaiser et al., 2013; Südkamp et al., 2018).

Although there is sparse research on experienced and novice teachers’ noticing and reasoning about deep cues, Jacobs et al. (2010) explored how teachers with varying levels of experience notice and reason about students’ mathematical understanding in on-the-fly assessments. Even when being explicitly prompted to focus on student understanding, novice teachers failed to point to specific evidence; by contrast, the large majority of experienced teachers was able to provide evidence regarding students’ level of understanding.

Building on the finding of Seidel et al. (2020) that experienced teachers were partially more accurate than novice teachers at diagnosing student profiles, Schnitzler et al. (2020) further explored the reasoning of novice teachers in terms of cues regarding student behavior (e.g., hand-raisings). Using epistemic network analyses (ENA; Shaffer, 2017)—a method that is designed to explore epistemic processes, such as teachers’ reasoning (e.g., Bauer et al., 2020; Farrell et al., 2022)—Schnitzler et al. (2020) explored the reasoning of novice teachers from the sample of Seidel et al. (2020) regarding different indicators for student engagement (i.e., behavioral, cognitive, emotional, knowledge-related, and confidence-related indicators; see Rimm-Kaufman et al., 2015) across different student profiles. The findings indicated that generally, the novice teachers mainly focused on the intensity of student engagement in terms of well-observable behavioral cues (e.g., students’ hand-raising), which can be considered surface cues. In addition, novice teachers sometimes referred to the content of students’ engagement (e.g., students’ quality of verbal contributions) in their reasoning, which might be considered as ranging between surface and deep cues (see Jacobs et al., 2010). Deep cues that were more inferential—for example, cues regarding students’ cognitive (e.g., inattention) or emotional engagement (e.g., interest), as well as students’ confidence (e.g., certainty in providing answers)—were hardly included in novices’ reasoning. In terms of judgment accuracy, the study found that novice teachers with comparably high accuracy in judging student profiles focused not exclusively on behavioral cues and related cues in ways that differentiated between student profiles with similar patterns of cues. For example, to identify the underestimating student profile, novice teachers with high accuracy focused on behavioral cues indicating the intensity of engagement (e.g., raising hands) and additionally considered the content of students’ engagement (e.g., students’ quality of verbal contributions)—which facilitated distinguishing the underestimating student profile, for example, from the struggling student profile. By contrast, novice teachers with low judgment accuracy seemed to miss or misinterpret those cues that facilitated successful differentiation between similar student profiles.

However, Schnitzler et al. (2020) focused on the analysis of novice teachers’ diagnostic reasoning and, therefore, did not include the experienced teachers from the study of Seidel et al. (2020) in their investigations. Thus, experienced teachers’ reasoning when diagnosing student profiles remained to be explored, to better understand how experienced teachers might differ from novice teachers in their reasoning when diagnosing latent student characteristic profiles based on student cues.

## 2 The present study

The present study investigates differences between novice and experienced teachers’ judgment accuracy and their diagnostic reasoning when asked to diagnose consistent and inconsistent student profiles (Seidel, 2006; Kosel et al., 2020). In doing so, we included the novice teachers investigated by Seidel et al. (2020) and Schnitzler et al. (2020) and the experienced teachers from Seidel et al. (2020), while adding additional experienced teachers to the sample in order to achieve comparable group sizes in the two subsamples. Because of the increased subsample of experienced teachers, we decided to investigate the replicability of the findings regarding the difference in novice and experienced teachers’ judgment accuracy in diagnosing student profiles. In our study, teachers’ judgment accuracy refers to their performance in accurately assigning five student characteristic profiles (i.e., strong, struggling, overestimating, uninterested, and underestimating students) to five videotaped students, whose characteristic profiles were empirically determined in advance.

However, the main attention of our research was set on exploring the reasoning of experienced teachers in comparison to the reasoning of novice teachers when diagnosing student profiles because, to our knowledge, this question has not been explored in research thus far. In our study, teachers’ diagnostic reasoning is characterized by student engagement cues coded in teachers’ written explanations of their diagnostic judgments. We explore novice and experienced teachers’ diagnostic reasoning about cues regarding student engagement using the method of ENA (Shaffer, 2017), which is a powerful tool to explore the reasoning about cues regarding student engagement when diagnosing student profiles. In doing so, the study aimed to gain insights into how professional experience influences teachers’ diagnosing of student profiles, which might offer valuable implications for supporting educational practice and designing targeted training for teacher education.

Teachers’ judgment accuracy and diagnostic reasoning might differ systematically across varying student characteristics profiles, which may result, for example, in a higher or lower overall judgment accuracy across all student profiles. In addition, investigating novice and experienced teachers’ diagnosing of individual student profiles (i.e., strong, struggling, overestimating, uninterested, and underestimating students) can indicate which student profiles are most challenging to diagnose and what might be reasons for performance differences between novice and experienced teachers’ diagnosing. The two research questions addressed in our research are:

**RQ1**: Are there systematic differences between novice and experienced teachers (a) in their overall judgment accuracy across student profiles and (b) in their judgment accuracy regarding individual student profiles?

Seidel et al. (2020) report evidence with a smaller sample of experienced teachers suggesting that experienced teachers tend to have an advantage over novices when diagnosing student profiles. Over time, experienced teachers have encountered a wide variety of cues and common cue patterns (Carter et al., 1988; Boshuizen et al., 2020) and have thereby developed a fine-grained professional vision (Gegenfurtner et al., 2022). Therefore, we hypothesize that, compared to novice teachers, experienced teachers show (a) a higher overall judgment accuracy when diagnosing student profiles and (b) a higher judgment accuracy regarding individual student profiles.

**RQ2**: What combination of cues do experienced teachers use in their reasoning when diagnosing student profiles and is there a systematic difference compared to novice teachers in (a) the overall reasoning across different student profiles and (b) the reasoning regarding individual student profiles?

Also for this exploratory research question, we assumed that experienced teachers’ professional vision facilitates their diagnostic reasoning, possibly resulting in a higher variety and a higher number of cues—including deep cues—compared to novice teachers, who were found to refer primarily to surface cues regarding student engagement when diagnosing student profiles (Schnitzler et al., 2020).

## 3 Methods

### 3.1 Participants

The sample consisted of *N* = 43 participants and included *n* = 24 novice teachers (female = 55%) enrolled in a university bachelor’s degree program to become secondary mathematics teachers and *n* = 19 in-service mathematics teachers (female = 64%) with a mean teaching experience of *M* = 10.92 years (*SD* = 9.11, *range* = 1.5–25.0 years). The subsample of novice teachers was the same as explored in Schnitzler et al. (2020) and Seidel et al. (2020); the subsample of experienced teachers was extended by 11 participants compared to the study of Seidel et al. (2020).

### 3.2 Procedure and materials

The present study was conducted under the Ethical Principles of Psychologists and the 2017 Code of Conduct of the American Psychological Association (American Psychological Association, 2017). Participants were assured that their data would be used following privacy policies and analyzed for scientific purposes only. Participants provided informed consent before participation.

The experiment was conducted in the laboratory, with only one participant at a time. Participants were seated in front of a computer and the experiment was conducted in the experimental computer environment Enterprise Feedback Suite Survey 22.2 (Tivian, 2022). First, participants were given a short theoretical introduction to each of the student characteristics under study: cognitive ability, interest, prior knowledge, and self-concept as well as their within-person interplay in strong, struggling, overestimating, underestimating, and uninterested student profiles.

**Figure 1:**
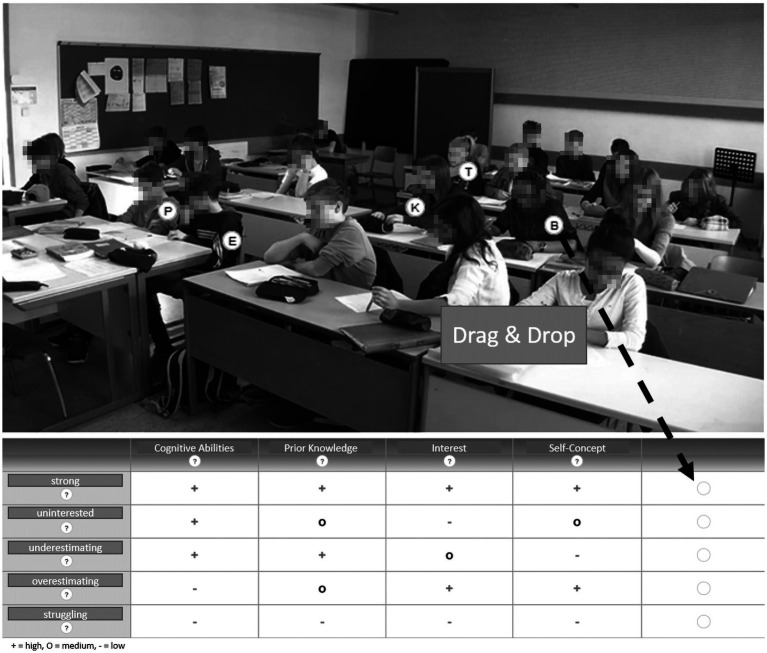
Example screenshot of the video clip and final drag & drop judgment task after observing the video clip with the labeled students.

After the introduction, participants watched a short video (2:30 min) of a lesson to familiarize themselves with the lesson topic and the classroom environment. Next, participants were instructed to carefully observe an 11-min video stimulus and diagnose student profiles afterward (see Figure 1). The 11-min video showed an eighth-grade geometry introductory lesson from a German high school. The video clip was recorded in the context of a previous video study on teacher-student interactions in classrooms and showed natural student behavior as it was videotaped in a real classroom situation (Seidel et al., 2016). Each target student was labeled with a random letter (B, E, K, P, T) throughout the video clip.

The labeled students in the video represented the strong, struggling, uninterested, overestimating, and underestimating student profiles. The student profiles were empirically determined using latent clustering in prior research by Seidel (2006) as well as Huber and Seidel (2018). This person-centered and latent clustering-based research aimed to explore homogenous subgroups of students, each distinctly characterized by a unique combination of cognitive characteristics (such as prior knowledge) and motivational-affective characteristics (e.g., self-concept). For instance, a specific student profile is assigned to students who demonstrate both high self-concept and substantial prior knowledge, categorizing them as strong students. This group is statistically differentiated from others, notably those with high self-concept yet limited prior knowledge, who are classified as overestimating students. However, it is important to recognize that the accuracy of these student profiles is dependent on the precision and robustness of the underlying research methods and instruments used and that the student profiles studied cannot be treated as objective truths. Latent clustering assigns students to student profiles based on the probability of them belonging to a specific homogenous subgroup including assignment errors (Spurk et al., 2020).

### 3.3 Measures

#### 3.3.1 Judgment accuracy

The correct judgment of a student was based on its match to the corresponding student profile. To perform the judgment after observing the video clip with the labeled students (the letters were unconnected to the profiles), participants were prompted to drag and drop the letters into a table, corresponding to their judgment of the student profile (see Figure 1). In case they were uncertain, they were also able to assign an additional, alternative profile. For each student profile, participants were assigned an accuracy score: A score of 0 represented an incorrect diagnosis. If a teacher first assigned an incorrect profile but stated the correct profile in their alternative choice, they received 0.5 points. Teachers received a score of 1 for a correct diagnosis. Overall, participants’ cumulative scores could range from a score of 0 (no correct judgments) to a score of 5 (all judgments correct).

#### 3.3.2 Reasoning

To analyze the reasoning of experienced and novice teachers, we coded their open-ended responses to a question that asked the participants about the diagnostically relevant cues they had observed and used to judge student profiles. This question was asked for each of the five target student profiles separately. To code the written responses, we used a fine-grained coding scheme developed by Schnitzler et al. (2020; building on research on student engagement, e.g., Rimm-Kaufman et al., 2015), consisting of five categories of codes: (1) knowledge (e.g., high quality of verbal contributions, problems with comprehension), (2) behavioral engagement (e.g., active participation, frequent hand-raising), (3) cognitive (e.g., student is attentive), (4) emotional engagement (e.g., student is interested or bored), and (5) student confidence (student is certain and uncertain). Overall, the coding scheme included these 5 categories and 26 corresponding sub-codes, as shown in Table 1. Two researchers coded the open-ended responses inductively following the coding scheme and reached a sufficiently high interrater agreement for the sample of novice teachers (Cohen’s *κ* = 0.93) and for the sample of experienced teachers (Cohen’s *κ* = 0.89).

**Table 1:** Coding scheme for student behavioral cues.

Category	Codes
Knowledge	High quality of verbal contributions
	Low quality of verbal contributions
	Understanding of topic
	Problems with understanding
	Helps classmates
	Receives help
Behavioral	Active participation
	No participation
	Frequent hand-raisings
	No or only few hand-raisings
	Fast working
	Slow working
	Following gaze
	Digressive gaze
	Interacts with classmates
	Does not interact with classmates
	Inconspicuous
	Otherwise involved
Cognitive	Attentive
	Inattentive
	Concentrated
Emotional	Interested
	Uninterested
	Bored
Confidence	Certain
	Uncertain

Adapted from Schnitzler et al. (2020).

### 3.4 Data analysis

#### 3.4.1 RQ1: ANOVA of teachers’ judgment accuracy

To examine the judgment accuracy of experienced and novice teachers, the distribution of their overall judgment accuracy scores was examined descriptively. Second, a 5×2 factorial ANOVA was used to examine differences in judgment accuracy across five student profiles (factor 1) and different levels of professional experience (factor 2) (RQ1a). Then a *post hoc* analysis was conducted using the Benjamini-Hochberg procedure to control for multiple comparisons. The Benjamini-Hochberg procedure is a method used to control the false discovery rate when conducting multiple comparisons (Agresti, 2007). The false discovery rate is the expected proportion of false positives among all significant results. Unlike the traditional Bonferroni correction, which controls the familywise error rate and can be overly conservative, the Benjamini-Hochberg method provides a balance between reducing the risk of Type I errors (false positives) and maintaining statistical power (Agresti, 2007). In our analysis, we used the Benjamini-Hochberg procedure to adjust the *p*-values obtained from pairwise *t*-tests comparing the judgment scores for each profile between novice and experienced teachers (RQ1b). We performed these tests to determine if there were significant differences in judgment scores for each profile based on the teacher’s experience level. Statistical analyses were performed using Python and the Pandas library (McKinney, 2010).

Upon conducting diagnostic checks for the ANOVA, we found that homogeneity of variances was maintained, as affirmed by Levene’s test (*p* > 0.05). No outliers were identified in the judgment scores, with the definition for an outlier being *z* > 3 (Grubbs, 1969). In addition, we verified the assumption of independence of observations. This confirms that each data point in our data set is independent of the others, ensuring the validity of the conclusions drawn from our analysis. However, the Shapiro–Wilk test showed a non-normal distribution of residuals, violating the normality assumption and implying potential skewness or heavy-tailed residuals. Despite this violation, two-factor ANOVAs’ robustness against such a deviation allowed us to remain within the parametric analysis design (Edwards, 1993).

#### 3.4.2 RQ2: ENA of teachers’ reasoning

To investigate novice and experienced teachers’ diagnostic reasoning, we used the ENA method (Shaffer, 2017) to explore the cues that were coded in the participants’ written responses. The general data processing of the ENA and the decisions to be made for the analysis are explained in the following (for an extended tutorial on ENA see Shaffer et al., 2016).

As a basis for the network model, the ENA algorithm accumulates co-occurrences of elements in coded data (e.g., observable cues coded in written responses). For doing so, it is required to specify how and for which units ENA should accumulate co-occurrences of codes: Our data consisted of participants’ reasoning regarding individual student profiles, recorded in one short written response per student profile. Because of (a) the shortness of the responses and (b) the task to reason about a diagnostic judgment, we assumed that each written response intended to create a coherent overall meaning and, thus, that all codes within a written response should be considered as interconnected; thus, we decided to set the “window” for accumulating the data (referred to as “stanza”) to the setting of “whole conversation,” meaning that co-occurrences of codes in our data were initially accumulated for each written response (alternatively, ENA allows, for example, to use a “moving window” setting to account for temporality in the data). We used a weighted summation of the codes (instead of a binary summation), accounting for varying frequencies of codes (i.e., cues) in the data that we considered as indicating how important participants considered different cues. Because the written responses were interdependent for each participant, who further belonged to the group of either novice or experienced teachers, we set the unit for analysis to “participant” and then further accumulated the participants per subsample group (i.e., novice and experienced teachers).

The ENA algorithm accumulates the coded data for each stanza (e.g., written response per student profile) and each unit of analysis (e.g., participants grouped into subsamples of novice and experienced teachers) into cumulative adjacency matrices that are further converted into adjacency vectors in a high-dimensional space. The adjacency vectors are then spherically normalized to control for differences in the overall amount of data per unit of analysis (e.g., length of written responses per participant), thereby transforming frequencies of co-occurrences to relative frequencies of co-occurrences. To facilitate interpretation and visualization of the normalized adjacency vectors, ENA performs a singular value decomposition: It rotates the original high-dimensional space such that the rotated space provides a reduced number of dimensions that account for the maximum variance in the data. The resulting multidimensional network model can then be depicted as two-dimensional network graphs. Per default, the graphs align the dimension with the highest amount of explained variance with the x-axis and the dimension with the second highest amount of explained variance with the *y*-axis. However, instead of using this default setting, we used the option of “means rotation,” which is recommended for comparing differences between two groups (e.g., novice and experienced teachers): The means rotation identifies the dimension with the highest systematic variance in explaining the differences between two selected groups and aligns this dimension with the *x*-axis of the network graph.

For every unit (e.g., participants), the ENA algorithm identifies at which point the corresponding normalized adjacency vector is located. For grouped units (e.g., participants grouped as novice and experienced teachers), the point representing the overall group can be considered a group mean. When using means rotation, the group means or the selected two groups are aligned with the *x*-axis. To facilitate interpretation, we consistently positioned novice teachers on the left and experienced teachers on the right for all network graphs.

In the two-dimensional network graphs, the coded cues in teachers’ responses are represented by gray nodes, with the size of the gray nodes referring to the relative frequency of their occurrences. The location of the nodes is relative to the normalized vectors for each unit: In our network graphs, this means, for example, that nodes (i.e., cues in teachers’ reasoning) that are close to one of the group means (e.g., cues positioned rather left in the network space, toward the novice teachers’ group mean) are more typically associated with the that group than with the other group whose group mean is more distant (e.g., experienced teachers on the right).

The colored edges in the network graphs refer to the relations (i.e., co-occurrences) of cues, with thickness indicating the strength of relations (i.e., the relative frequency of co-occurrences). Weak relations were not shown in our network graphs to facilitate the interpretability of the networks (the minimum edge weight was set to 0.06). For group comparisons, ENA creates a set of three related network graphs respectively: In our analysis, one network graph depicts the novice teachers’ reasoning, one network graph depicts the experienced teachers’ reasoning, and a comparison graph depicts only the differences between the novice and experienced teachers’ reasoning.

To explore (RQ2a) novice and experienced teachers’ reasoning across different student profiles, we initially compared the network graphs as specified above, accumulating co-occurrences of cues coded in the written responses per participant and then per group of novice and experienced teachers. To explore (RQ2b) novice and experienced teachers’ reasoning about each student profile in more detail, we filtered the written responses that addressed the individual student profiles and then performed the same analysis for each student profile.

In addition to performing a qualitative interpretation of the network graphs, we statistically tested group differences between novice and experienced teachers’ reasoning, using one independent-samples *t*-test for each comparison. For RQ2a, the alpha level was set to *α* = 0.05. For RQ2b, we controlled the false discovery rate when conducting multiple comparisons by using Bonferroni-adjusted alpha levels of *α* = 0.01 (*α* = 0.05/5). We created the network graphs with the ENA online tool.^1^

## 4 Results

### 4.1 RQ1: Teachers’ judgment accuracy

#### 4.1.1 RQ1a: Teachers’ overall judgment accuracy

**Figure 2:**
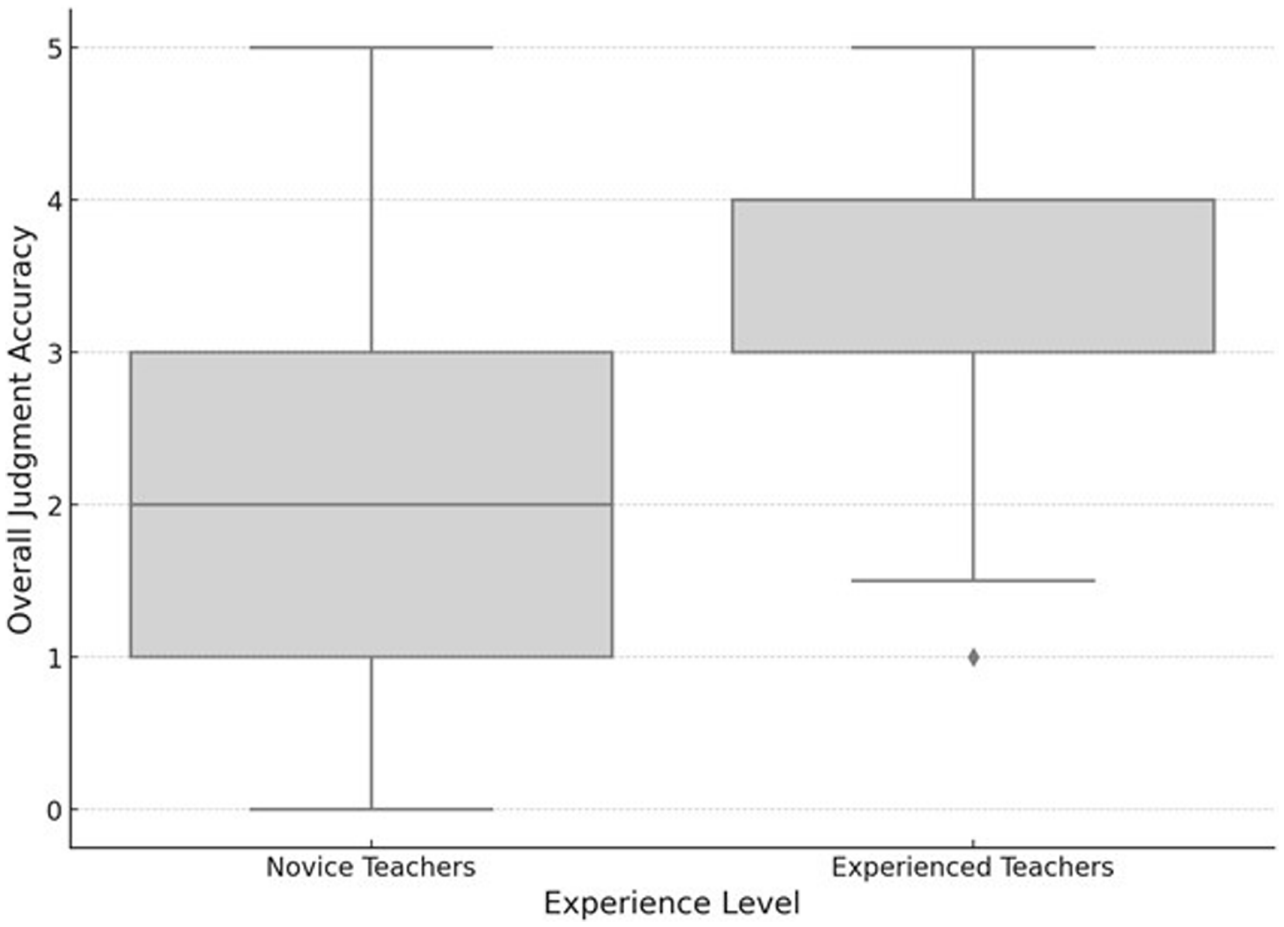
Overall judgment score across experience levels.

The primary goal of our first research question is to identify potential systematic differences between novice and experienced teachers with regard to the accuracy of their judgments. Specifically, we aim to (a) assess their overall accuracy in assessing different student profiles, and (b) examine the accuracy of their judgments for each individual student profile. Descriptively, we found that experienced teachers generally had a higher overall judgment score (*M* = 3.47; *SD* = 1.26), compared to novice teachers (*M* = 2.42; *SD* = 1.62). However, the standard deviations indicate substantial variability within both groups. Figure 2 presents a boxplot visualization of the overall judgment accuracy, highlighting a higher median score for experienced teachers. Additionally, the boxplot suggests a slightly larger range of scores for novice teachers, implying greater variability in their overall judgment scores.

#### 4.1.2 RQ1b: Teachers’ judgment regarding individual student profiles

**Figure 3:**
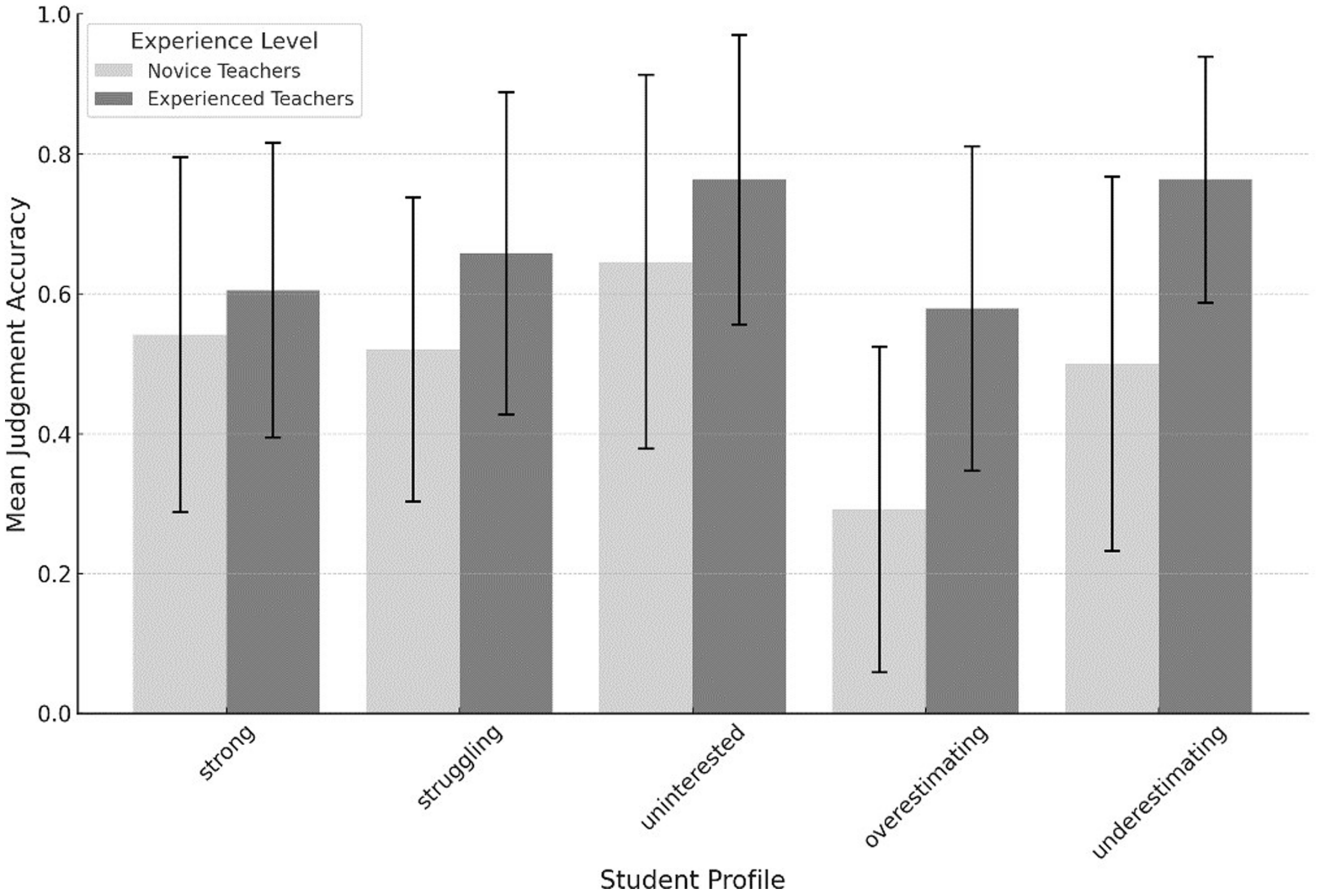
Mean judgment accuracy is separated for each student profile across experience levels.

In a more granular examination of judgment accuracy, we differentiated the analysis by individual student profiles (Figure 3). We observed systematic variations between experienced and novice teachers in their judgment (sorted from best to worst judgment scores): When judging the underestimating profile, experienced teachers had a mean score of 0.76 (*SD* = 0.39), whereas novice teachers had a mean score of 0.50 (*SD* = 0.44). In the uninterested profile, experienced teachers had a mean judgment score of 0.76 (*SD* = 0.42) in contrast to the novice teachers’ mean score of 0.65 (*SD* = 0.48). For the struggling profile, the mean judgment score was 0.66 (*SD* = 0.44) for experienced teachers and 0.52 (*SD* = 0.48) for novice teachers. For the overestimating profile, experienced teachers had a mean judgment score of 0.58 (*SD* = 0.42), while novice teachers had a mean score of 0.29 (*SD* = 0.44). In judging the strong profile, experienced teachers demonstrated a mean score of 0.61 (*SD* = 0.43) compared to novice teachers’ mean score of 0.54 (*SD* = 0.46).

In sum, it appears that both novice and experienced teachers consistently rate the uninterested and underestimating student profiles with the highest mean scores. However, when it comes to assessing the strong and overestimating profiles, experienced teachers exhibit superior judgment accuracy. In the next step, we analyze if these systematic variations are statistically significant.

A 5×2 factorial ANOVA (see Table 2) was performed to probe the differences in judgment accuracy, with the five distinct student profiles and varying levels of professional experience serving as the two factors under consideration. The main effect of student profile type was not significant (*F*(4, 90) = 0.89, *p* = 0.47, *η*^2^ = 0.04), indicating that there was no significant difference in judgment scores across student profiles when teacher experience was not taken into account. However, the main effect of teacher experience level was significant (*F*(1, 90) = 3.93, *p* = 0.05, *η*^2^ = 0.04), indicating a significant difference in judgment scores between novice and experienced teachers. The interaction effect between student profile type and teacher experience was not significant, *F*(4, 90) = 0.94, *p* = 0.45, *η*^2^ = 0.01, indicating that the effect of student profile type on judgment scores did not differ significantly between novice and experienced teachers.

**Table 2:** 5×2 factorial ANOVA: differences in judgment accuracy.

Source of variation	SS	df	MS	F	*p*	*η*^2^
Student profile	0.17	4	0.04	2.26	0.47	0.04
Experience level	0.16	1	0.16	3.93	0.05	0.04
Student profile x Experience level	0.16	4	0.04	0.94	0.45	0.01
Residual	17.36	90	0.19			

Following the ANOVA, we conducted a *post hoc* analysis using multiple t-tests to compare the judgment score of novice and experienced teachers for each student profile. To adjust for the increased risk of Type I error associated with multiple comparisons, we applied the Benjamini-Hochberg correction procedure. We found that all adjusted *p*-values exceeded the conventional significance level of 0.05. This suggests that, when accounting for the multiplicity of tests performed, there were no statistically significant differences in the judgment scores of novice and experienced teachers within the different student profiles.

### 4.2 RQ2: Teachers’ reasoning

#### 4.2.1 RQ2a: Teachers’ reasoning across different student profiles

In order for tackle our second research question, we analyzed the coded open-ended questions. The responses provide insights into the specific student cues that teachers relied on when deducing underlying student profiles based on their observations. Table 3 displays the 15 most frequently stated behavioral cues, separated by experience level. On average, experienced teachers indicated 5.32 cues, and novice teachers 2.52 cues. The overall frequencies of cues and the frequencies of individual cues suggest that, compared to novice teachers, experienced teachers generally consider more and also a greater variety of cues. This finding is further elaborated in the following, integrating the descriptive results with the interpretation of the epistemic networks of novice and experienced teachers’ diagnostic reasoning.

**Table 3:** The 15 most frequently utilized student cues, sorted by category and separated by experience level.

Category	Cue	ET (%)	NT (%)	ET (freq.)	NT (freq.)
Knowledge	High-quality contributions	36%	27%	35	32
	Low-quality contributions	35%	25%	34	30
	Understand the topic	23%	7%	22	9
	Problems to understand the topic	17%	6%	17	8
Behavioral	A lot of hand-raisings	32%	25%	31	30
	No / few hand-raisings	27%	29%	30	34
Cognitive	Attentive	34%	20%	33	24
	Inattentive	29%	11%	28	14
Emotional	Interested	24%	11%	23	13
	Uninterested	15%	4%	15	5
Confidence	Certain	43%	29%	41	34
	Uncertain	30%	13%	29	16

ET, Experienced Teachers; NT, Novice Teachers.

**Figure 4:**
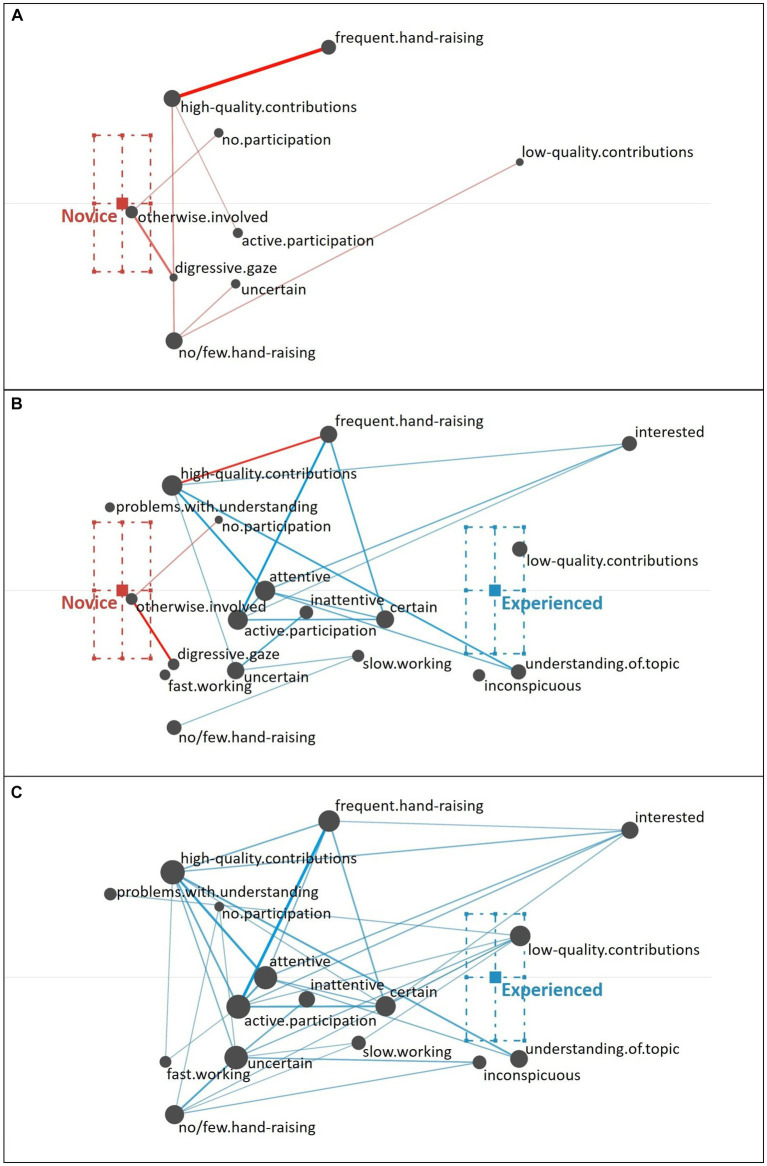
Epistemic network of teachers’ reasoning across the five different student profiles from **(A)** novice teachers and **(C)** experienced teachers, with the **(B)** comparison network showing only the differences between novice and experienced teachers’ reasoning across all five student profiles. Gray nodes correspond to cues, with node size referring to the relative frequency of their occurrence; colored edges refer to the relations (i.e., co-occurrences) of cues, with thickness indicating the strength of relations.

Using ENA, we can examine not only the frequency (i.e., occurrences) of individual cues, represented by the size of the gray nodes, but additionally the strength of relations (i.e., co-occurrences) of cues, which is represented by the thickness of the colored edges. Cues positioned toward the left, are more typically associated with novice teachers and cues positioned toward the right are more typically associated with experienced teachers. As described by Schnitzler et al. (2020), novice teachers primarily focus on well-observable behavioral cues (i.e., surface cues; e.g., a lot of hand-raising, digressive gaze) and additionally include some cues that refer to students’ knowledge and comprehension of the topic (see Figure 4A). Looking at the relations of cues, novice teachers typically seem to combine these two types of cues (e.g., a lot of hand-raising with high-quality contributions; few hand-raising with low-quality contributions).

By comparison, experienced teachers’ reasoning (see Figure 4C) across all student profiles indicates that experienced teachers consider a broad variety of student engagement cues when diagnosing student profiles, including well-observable behavioral cues (i.e., surface cues; e.g., a lot of hand-raising) but also motivational-affective cues that are partially rather latent and require some degree of inference (i.e., deep cues; e.g., uncertain). In addition, these various cues show to be well interrelated, which suggests that experienced teachers use a variety of cues in their reasoning and do so across different student profiles.

The difference between novice and experienced teachers’ reasoning is further highlighted in the comparison graph (see Figure 4B), which shows a subtraction of the experienced and the novice teachers’ reasoning networks: The comparison graph further highlights the observation that, overall, novice teachers related fewer and less varying cues compared to experienced teachers who related a broad variety of cues in their reasoning. This observation is supported by the frequencies of individual cues included by novice and experienced teachers in their reasoning (see Table 3).

The difference between novice teachers (position of the mean on the *x*-axis: *M* = −0.23, *SD* = 0.10) and experienced teachers (position of the mean on the *x*-axis: *M* = 0.29, *SD* = 0.09) in their reasoning across all five student profiles was statistically significant, *t*(42.30) = −19.14, *p* < 0.01, Cohen’s *d* = 5.68.

Thus, the findings indicate that there are substantial differences in experienced and novice teachers’ reasoning when diagnosing student profiles.

#### 4.2.2 RQ2b: Teachers’ reasoning about individual student profiles

The differences between novice and experienced teachers regarding their utilization of cues and relations drawn between cues can be further differentiated per student profile. Specific differences in novice and experienced teachers’ reasoning when diagnosing the individual student profiles are illustrated in the following.

**Figure 5:**
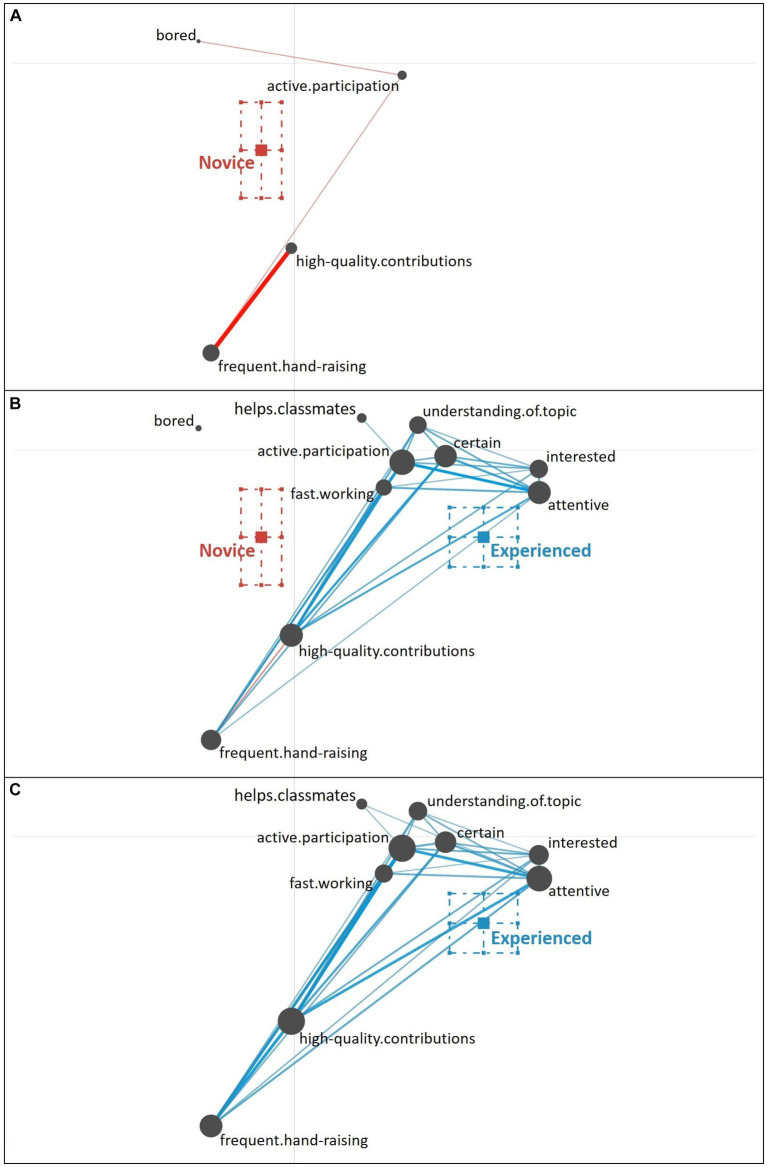
Epistemic network of teachers’ reasoning regarding the strong student profile from **(A)** novice teachers and **(C)** experienced teachers, with the **(B)** comparison network showing only the differences between novice and experienced teachers’ reasoning. Gray nodes correspond to cues, with node size referring to the relative frequency of their occurrence; colored edges refer to the relations (i.e., co-occurrences) of cues, with thickness indicating the strength of relations.

**Strong Student**. When diagnosing the strong student, novice teachers (see Figure 5A) focused especially on behavioral cues (i.e., surface cues), such as the student’s frequent hand-raising. Novice teachers associated this behavior with the student’s active participation and occasional signs of boredom, as well as the student’s high quality of contributions (i.e., cue about the student’s knowledge).

As indicated by the network of experienced teachers’ reasoning (see Figure 5C) and the comparison graph (see Figure 5B), experienced teachers considered and related various behavioral and motivational-affective cues: These cues included cues that are not directly observable but involved some degree of inference (i.e., deep cues) on the side of the teacher, such as the student being interested and certain; however, the teachers related these cues to directly observable behavioral cues (e.g., a lot of hand-raising) as well as cues about the student’s knowledge (e.g., high quality of contributions, understanding of the topic).

The difference between novice teachers’ reasoning (position of the mean on the *x*-axis: *M* = −0.09, *SD* = 0.12) and experienced teachers’ reasoning (position of the mean on the *x*-axis: *M* = 0.49, *SD* = 0.18) regarding the strong student profile was statistically significant, *t*(30.26) = −11.72, *p* < 0.01, Cohen’s *d* = 3.77.

**Figure 6:**
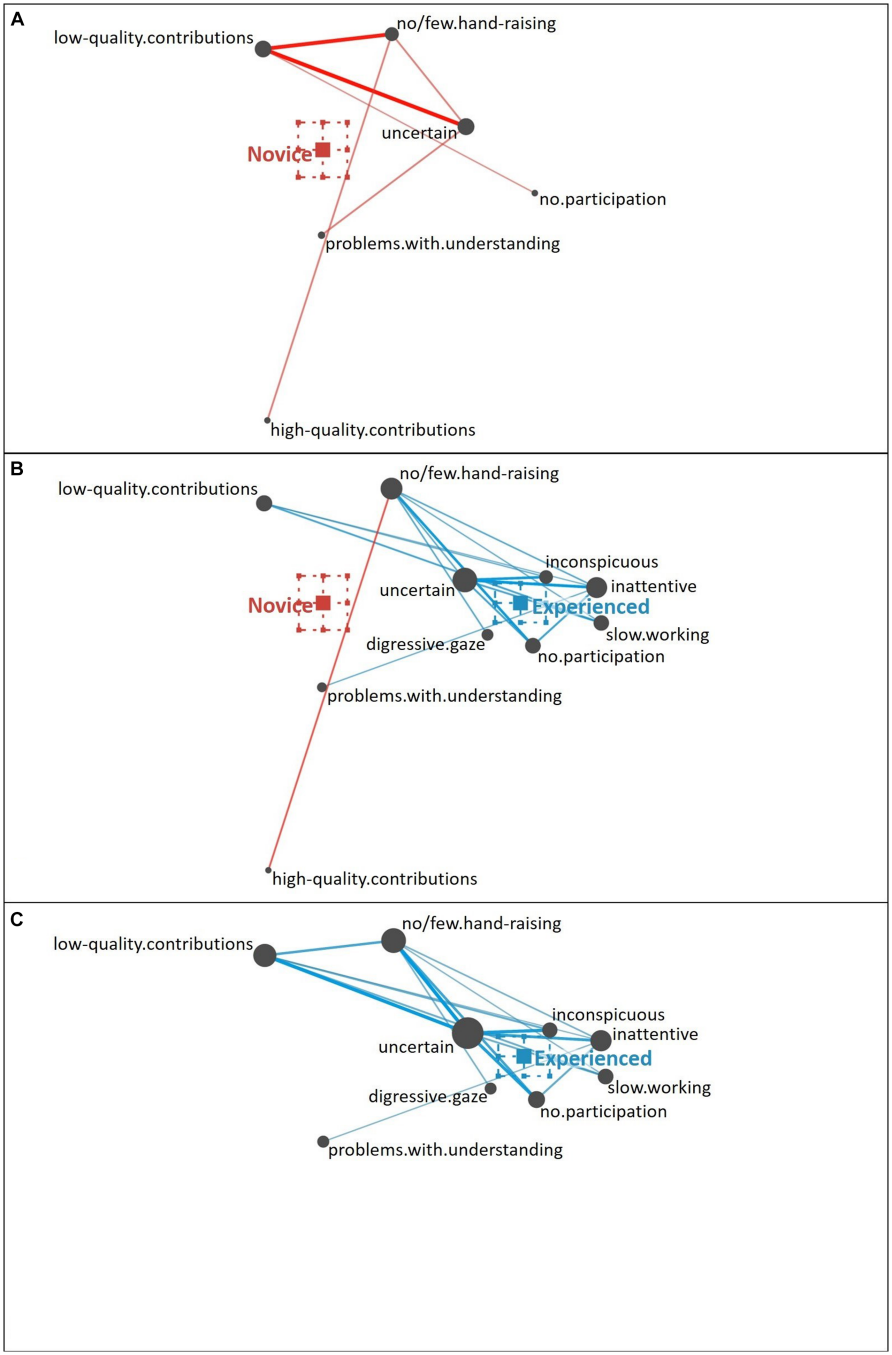
Epistemic network of teachers’ reasoning regarding the struggling student profile from **(A)** novice teachers and **(C)** experienced teachers, with the **(B)** comparison network showing only the differences between novice and experienced teachers’ reasoning. Gray nodes correspond to cues, with node size referring to the relative frequency of their occurrence; colored edges refer to the relations (i.e., co-occurrences) of cues, with thickness indicating the strength of relations.

**Struggling Student**. Novice teachers characterized the struggling student (see Figure 6A) primarily based on the observation that the student exhibited hardly any hand-raising in combination with a low quality of their contributions and indications of uncertainty. Some novice teachers additionally pointed to a lack of participation and the student showing problems with understanding the topic, which is a combination of behavioral and knowledge-related cues as well. Interestingly, few novice teachers emphasized the high quality of the student’s contributions, indicating a misinterpretation of the cues, which might have resulted in an inaccurate judgment of the student profile (see Schnitzler et al., 2020).

This misinterpretation was not shown by experienced teachers (see Figure 6C). Other cues discussed by the novice teachers were also considered by experienced teachers, who additionally included further behavioral cues (e.g., digressive gaze, slow working style; i.e., surface cues; see also Figure 6B for the direct comparison of novice and experienced teachers). Interestingly, besides the cues that might easily be recognized as potentially problematic, experienced teachers also pointed to the student being quiet and inconspicuous as well as the student being inattentive.

The difference between novice teachers’ reasoning (position of the mean on the *x*-axis: *M* = −0.11, *SD* = 0.11) and experienced teachers’ reasoning (position of the mean on the *x*-axis: *M* = 0.29, *SD* = 0.11) regarding the struggling student profile was statistically significant, *t*(39.28) = −11.71, *p* < 0.01, Cohen’s *d* = 3.61.

**Figure 7:**
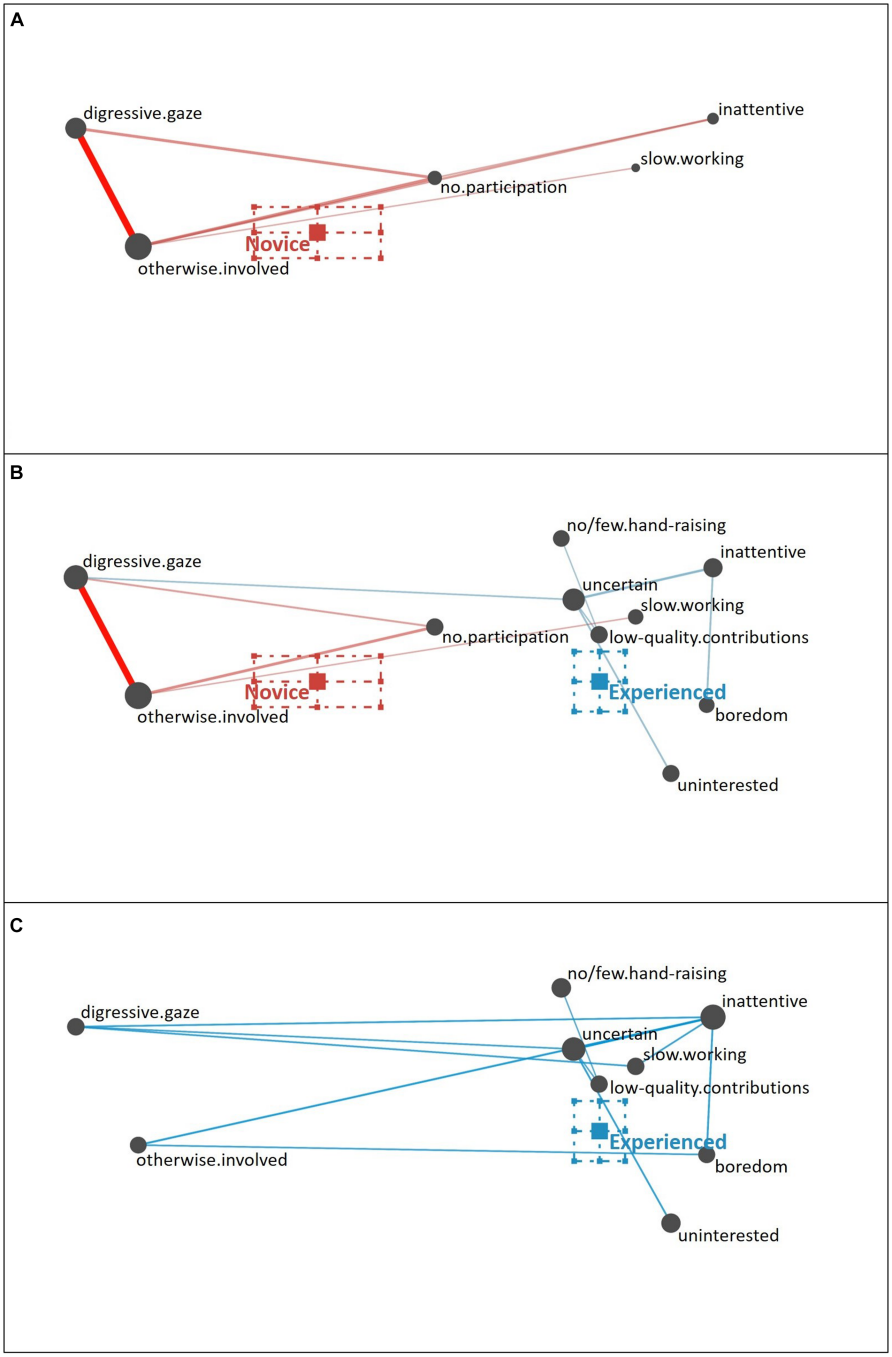
Epistemic network of teachers’ reasoning regarding the uninterested student profile from **(A)** novice teachers and **(C)** experienced teachers with the **(B)** comparison network showing only the differences between novice and experienced teachers’ reasoning. Gray nodes correspond to cues, with node size referring to the relative frequency of their occurrence; colored edges refer to the relations (i.e., co-occurrences) of cues, with thickness indicating the strength of relations.

**Uninterested Student**. Novice teachers described the uninterested student (see Figure 7A) as showing a digressive gaze in relation to being otherwise involved and not participating. Some novice teachers pointed out the student’s slow working style. In addition to these behavioral (i.e., surface) cues, some novice teachers recognized the student as inattentive, showing some initial capacity to notice some more inferential (i.e., deep) cues.

In comparison (see Figure 7B), the experienced teachers rather pointed to additional behavioral cues (e.g., no or few hand-raising) and also focused on more inferential motivational-affective cues (i.e., deep cues), such as the student being inattentive and uncertain (see Figure 7C).

The difference between novice teachers’ reasoning (position of the mean on the *x*-axis: *M* = −0.26, *SD* = 0.24) and experienced teachers’ reasoning (position of the mean on the *x*-axis: *M* = 0.18, *SD* = 0.08) regarding the uninterested student profile was statistically significant, *t*(31.06) = −8.55, *p* < 0.01, Cohen’s *d* = 2.33.

**Figure 8:**
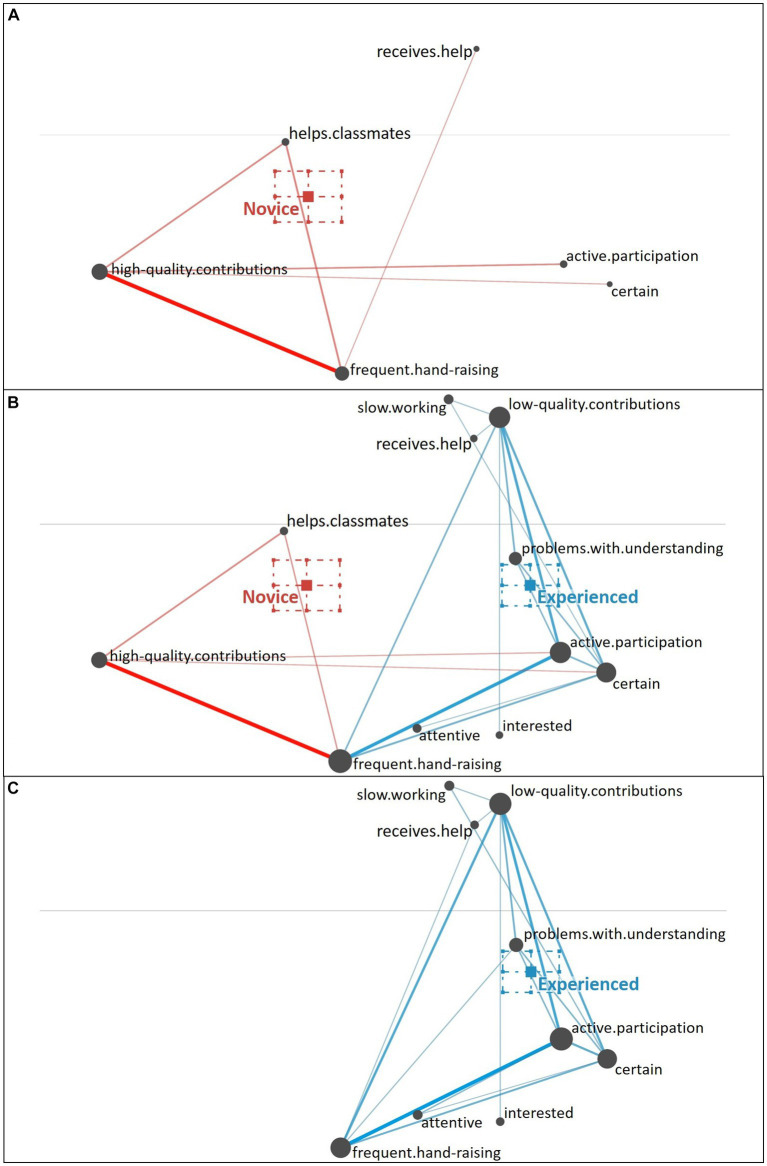
Epistemic network of teachers’ reasoning regarding the overestimating student profile from **(A)** novice teachers and **(C)** experienced teachers with the **(B)** comparison network showing only the differences between novice and experienced teachers’ reasoning. Gray nodes correspond to cues, with node size referring to the relative frequency of their occurrence; colored edges refer to the relations (i.e., co-occurrences) of cues, with thickness indicating the strength of relations.

**Overestimating Student**. The cues used by novice teachers to characterize the overestimating student (see Figure 8A) comprise of frequent hand-raising (behavioral cue), oftentimes combined with pointing to a high quality of contributions (knowledge-related cue) and sometimes with the student’s certainty and active participation in the lesson. Some novice teachers validated this impression with the observation that the student provides help or is asked for help by a second student (i.e., their seatmate), whereas few novice teachers interpreted the student talking to their seatmate differently, as seeking and receiving help. Overall, the cues involved in novice teachers’ reasoning are not specific to the overestimating profile but also applicable to the strong profile, which explains why many novice teachers diagnosed the overestimating student as a strong student (see Schnitzler et al., 2020).

The experienced teachers validated the behavioral (i.e., surface cues) cues of frequent hand-raising and active participation with further motivational-affective cues (i.e., deep cues) besides certainty, namely the attentiveness and interest displayed by the student (see Figure 8C). In contrast to the novice teachers (see Figure 8B), experienced teachers also did not misinterpret the quality of the student’s contribution as high but considered the quality of the student’s contribution as low. They also regarded the interaction of the student with their seatmate as seeking and receiving help. The experienced teachers’ reasoning was additionally backed up with further behavioral cues (i.e., slow working style) and knowledge-related cues (i.e., the student’s problems with understanding the topic), which illustrated a realistic overall assessment of the overestimating student’s skills.

The difference between novice teachers’ reasoning (position of the mean on the *x*-axis: *M* = −0.18, *SD* = 0.20) and experienced teachers’ reasoning (position of the mean on the *x*-axis: *M* = 0.40, *SD* = 0.15) regarding the overestimating student profile was statistically significant, *t*(39.78) = 10.71, *p* < 0.01, Cohen’s *d* = 3.24.

**Figure 9:**
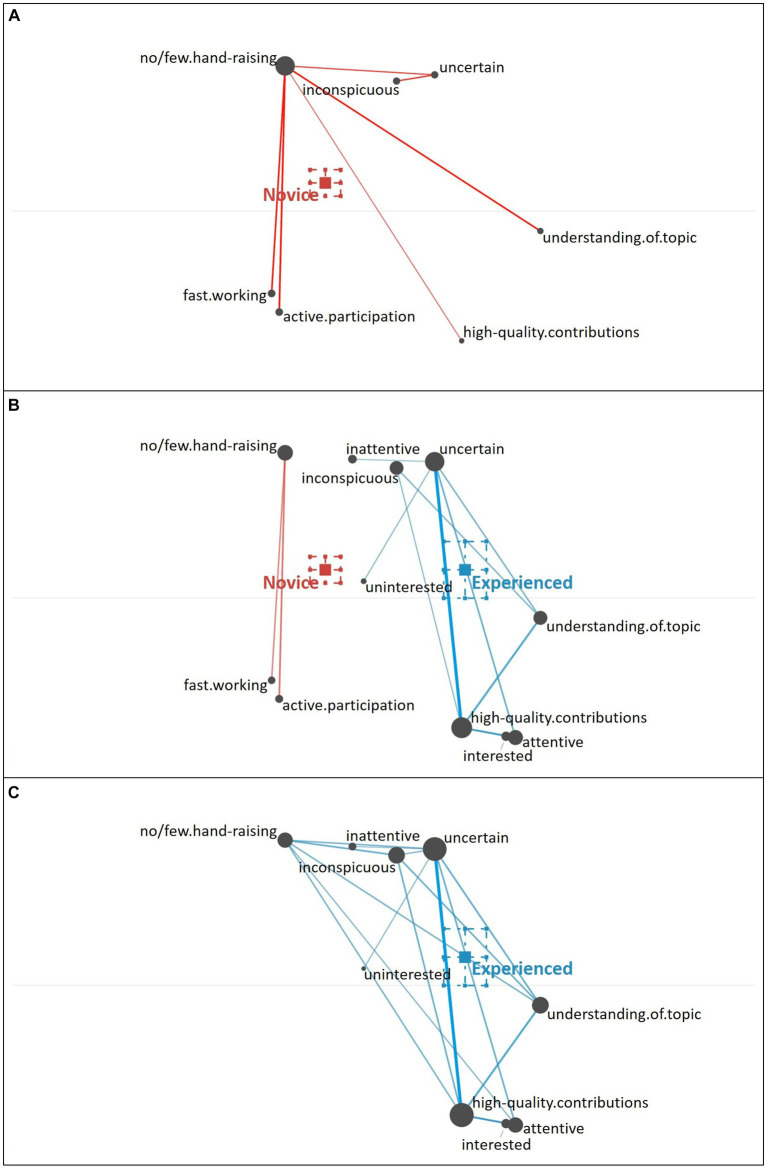
Epistemic network of teachers’ reasoning regarding the underestimating student profile from **(A)** novice teachers and **(C)** experienced teachers with the **(B)** comparison network showing only the differences between novice and experienced teachers’ reasoning. Gray nodes correspond to cues, with node size referring to the relative frequency of their occurrence; colored edges refer to the relations (i.e., co-occurrences) of cues, with thickness indicating the strength of relations.

**Underestimating Student**. When diagnosing the underestimating student, novice teachers again primarily focused on the behavioral cue of hand-raising (no or few hand-raising; i.e., surface cues) and tended to relate it to few additional cues out of three clusters (see Figure 9A): further behavioral cues (fast working style, active participation), knowledge-related cues (high quality of contributions, understanding of the topic), or a cluster of cognitive-behavioral cues expressing the student’s insecurity and caution (uncertain, quiet and inconspicuous).

The experienced teachers’ reasoning about the underestimating student (see Figure 9C) illustrates that they generally considered a higher number of cues and their relation to each other. Compared to the novice teachers (see Figure 9B), the experienced teachers focused less on the behavioral (i.e., surface) cues, but more on the knowledge-related cues and more inferential cues about the student’s cognitive and motivational-affective characteristics (e.g., the student’s attention and interest; i.e., deep cues).

The difference between novice teachers’ reasoning (position of the mean on the *x*-axis: *M* = −0.12, *SD* = 0.11) and experienced teachers’ reasoning (position of the mean on the *x*-axis: *M* = 0.31, *SD* = 0.13) regarding the underestimating student profile was statistically significant, *t*(34.23) = −11.11, *p* < 0.01, Cohen’s *d* = 3.52.

The analyses of teachers’ reasoning regarding the different student profiles showed that, compared to novice teachers, experienced teachers generally used a higher number of cues—of which a higher portion can be considered deep cues (e.g., about motivational-affective student characteristics)—and drew more relations between cues, thereby crafting a more comprehensive and robust reasoning than novice teachers. These observations were consistent across all individual student profiles.

## 5 Discussion

In this study, we delved into novice and experienced teachers’ (a) judgment accuracy and (b) reasoning about observable student cues when diagnosing student profiles with varying cognitive and motivational-affective characteristics. Five different student profiles were considered: three inconsistent types (overestimating, underestimating, and uninterested) and two consistent types (strong and struggling; Seidel, 2006; Kosel et al., 2020). Drawing on the framework of teacher professional vision, we assumed that when diagnosing student profiles, experienced teachers would make more accurate judgments than novice teachers. Over time, experienced teachers typically develop refined noticing and reasoning skills, based on their knowledge and experience of handling diverse classroom situations (Gegenfurtner, 2020; Wolff et al., 2021). Building on prior research (Schnitzler et al., 2020), we used the method of ENA (Shaffer, 2017) to analyze differences in novice and experienced teachers’ reasoning regarding the cues that they used for their diagnosing. The study adds two major findings to the research field: First, experienced teachers had a significantly higher overall judgment accuracy than novice teachers. Second, ENA showed that experienced and novice teachers differed significantly in their reasoning, both regarding the variety of considered cues and the relations drawn between the cues in their diagnosing.

### 5.1 The role of experience in teachers’ judgment accuracy

Consistent with our initial assumption, our results confirmed that, overall, experienced teachers were able to judge student profiles more accurately than novice teachers (RQ1a). This finding aligns with theoretical models of teacher judgment (e.g., Loibl et al., 2020), which emphasize that professional experience can have a substantial effect on judgment accuracy. Through practical experience, teachers elaborate and restructure their knowledge, thereby building higher-order knowledge representations that integrate declarative knowledge with prior experience (Boshuizen et al., 2020). Such prior experience includes encounters with a large number and variety of students. This exposure refines teachers’ cognitive prototypes of typical student profiles (Cantor and Mischel, 1977; Hörstermann et al., 2010; Papa, 2016). Using this enriched professional knowledge, experienced teachers have an improved professional vision (Seidel and Stürmer, 2014; Gegenfurtner, 2020) and thus, are better equipped to make accurate judgments when diagnosing student profiles.

However, the results of the post-hoc analysis indicated that the difference between experienced and novice teachers’ judgment accuracy was not significant at the level of individual student profiles (RQ1b). Examining the descriptive results, experienced teachers clearly had a higher mean judgment accuracy for each student profile compared to novice teachers. However, within both teacher groups, there was substantial variance in the judgment accuracy per student profile, as indicated by standard deviations. The results suggest that while many experienced teachers can accurately judge student profiles, a significant number also struggle to make accurate judgments. Other research has emphasized as well that despite the higher average judgment accuracy that is associated with increasing experience, there are also variations in experienced teachers’ judgment accuracy (Jacobs et al., 2010). The depth and quality of teachers’ knowledge might depend on their formal training, ongoing professional development, and individual experiences (e.g., regarding classroom challenges, student demographics, etc.). However, as pointed out by Schnitzler et al. (2020), also novice teachers can achieve high judgment accuracy when diagnosing student profiles. Variations in novice teachers’ knowledge might be explained as well by their individual education, initial practical experience in teaching, but also individual person characteristics that are not related to professional knowledge (e.g., self-concept and interest; Sorge et al., 2019). However, despite the variance in the judgment accuracy per student profile within both groups of experienced and novice teachers, experienced teachers (a) showed a higher baseline, higher mean, and lower standard deviation in their overall judgment accuracy (see boxplot in Figure 2) and (b) higher mean judgment accuracy per student profile. Thus, we consider the overall results of this study as support for the assumption that experienced teachers—through their elaborated knowledge and improved professional vision—can diagnose student profiles more accurately than novice teachers.

Interestingly, we found that experienced teachers performed particularly well in accurately judging some of the inconsistent student profiles, namely the uninterested and the underestimating student profile. This is in line with the finding of Südkamp et al. (2018), who initially assumed that making a holistic judgment based on inconsistent patterns of cues for cognitive and motivational-affective characteristics might result in lower accuracy; however, they empirically found that teachers in their study were not better at diagnosing consistent profiles compared to inconsistent profiles. Based on the evidence, we speculate that one factor in teachers’ development of cognitive prototypes concerning student profiles might be the frequency with which the student profiles occur in regular classrooms (i.e., exemplarity; see Fischer et al., 2022): As reported by Kosel et al. (2020), approximately 35% of students in a large sample of 9th-graders exhibited an underestimating profile. This finding was consistent in two different school subjects (i.e., mathematics and language arts), suggesting that the underestimating profile might be a common profile to observe in secondary school students; by contrast the frequency of other profiles varied across the two subjects. Experienced teachers, exposed to specific student profiles, might refine their cognitive prototypes of students over time, resulting in a refined professional vision and improved judgment accuracy in diagnosing the respective student profiles.

### 5.2 The role of experience in teachers’ diagnostic reasoning

To understand why experienced teachers achieve higher judgment accuracy in diagnosing student profiles, exploring their diagnostic reasoning can provide relevant insights into *how they reason* and *about which cues* they reason (Herppich et al., 2018; Loibl et al., 2020). Using ENA, we found (RQ2a) that experienced teachers, compared to novice teachers, used (a) generally a higher number of cues of which (b) a higher portion can be considered deep cues, for example, about motivational-affective student characteristics; moreover, experienced teachers (c) drew more relations between observed cues, thereby crafting a more comprehensive and robust reasoning than novice teachers, and did so (d) across all individual student profiles (RQ2b).

As already reported by Schnitzler et al. (2020), the novice teachers in our sample primarily referred to behavioral cues, such as hand-raising or active participation, and additionally considered cues about students’ knowledge in their reasoning. Especially the behavioral cues can be regarded as surface cues because they are focused on a directly observable level of student behavior (see Brunswik, 1955; Loibl et al., 2020). By contrast, as found in the present study, experienced teachers more frequently integrated deep cues into their reasoning, such as recognizing when a student is uncertain, inattentive, or interested. Such deep cues refer to a rather inferential level of the students’ cognitive and emotional engagement and are not necessarily directly observable (see Brunswik, 1955; Loibl et al., 2020). Experienced teachers seem to leverage surface cues (e.g., hand-raising) to infer deep cues (e.g., certainty), by using additional information to make inferences about not directly observable motivational affective student characteristics (e.g., interest). It might be assumed that these inferences require cognitive resources on the side of the teacher; however, the higher number of cues and the higher number of relations drawn between cues indicate that experienced teachers are very efficient in noticing and reasoning about cues. Thus, relations between observable cues on the surface and the deep level might be stored as part of teachers’ cognitive prototypes, which can be used as efficient heuristics when processing information during noticing and reasoning processes (see Kahneman and Klein, 2009; Boshuizen et al., 2020).

Such findings are consistent with previous expert-novice studies of teachers’ professional vision (Van Es and Sherin, 2010; Seidel and Stürmer, 2014; Gegenfurtner, 2020). These studies have collectively emphasized that experienced teachers generally outperform novices in both identifying (noticing) and interpreting (reasoning) cues that are relevant to teaching and learning (Gegenfurtner, 2020). Our findings are also consistent with the findings of Jacobs et al. (2010) that novice teachers struggle to identify and interpret deep cues (e.g., regarding students’ level of understanding), which a large majority of experienced teachers can identify and reason about. As in Jacobs et al. (2010) study, novice teachers in the present study may have also faced challenges in gaining sufficient insights from observing student behavior. Compared to experienced teachers, novice teachers might usually not have yet accumulated the required knowledge and experience for drawing more in-depth inferences from their observations (i.e., about deep cues) and thus, are more likely to remain on a surface level of reasoning (i.e., about surface cues).

We also speculate that the differences found between novice and experienced teachers’ reasoning in our study might partially trail back to novice and experienced teachers’ noticing of cues—which was, however, not investigated in the present study. Differences in novice and experienced teachers’ noticing processes have been examined by eye-tracking research which focused on how novice and experienced teachers observe and respond to student behavior. Experienced teachers typically exhibit an extended visual monitoring behavior, encompassing a larger subset of students (Kosel et al., 2021). Their monitoring behavior is more advanced, enabling them to gather detailed, nuanced information about diverse students in a short timeframe (Dessus et al., 2016; Verbert et al., 2016; Kosel et al., 2021, 2023). In contrast, as found by Dessus et al. (2016), novice teachers experience an increased cognitive load during monitoring students, resulting in a more limited focus on a smaller group of students. Research by Karst and Bonefeld (2020) shows that teachers’ judgment accuracy improves with a uniform distribution of attention across students, which emphasizes the impact of noticing processes on teachers’ judgment accuracy and, presumably, also their reasoning.

In addition to differences in the type of cues, our exploratory network analysis of teachers’ diagnostic reasoning indicated that experienced teachers drew more relations between observed cues, thereby crafting a more comprehensive and robust reasoning than novice teachers. For example, novice teachers’ reasoning about the overestimating student was not necessarily specific to the overestimating profile but indicated potential confusion with the strong profile. As reported by Schnitzler et al. (2020), their indeed tended to confuse the overestimating and the strong student profile. Besides their focus on behavioral cues (e.g., frequent hand-raising, active participation), one factor in novice teachers’ confusion was their misinterpretation of the quality of the student’s contributions (e.g., misinterpreting the overestimating student’s low-quality contributions as high-quality contributions). Moreover, by comparison, an additional difference is that expert teachers validated their observations about overestimating students by relating a broader number and variety of cues about cognitive and motivational-affective student characteristics in their reasoning. This pattern was observable across all different student profiles. As suggested by the lens model (Brunswik, 1955), experienced teachers might tend to correlate various cues, thereby checking the cues’ validity and making a probabilistic yet informed judgment. This is in line with research on expert decision-making in other areas than teaching (e.g., medicine), which indicates that domain experts (i.e., more experienced professionals in a specific domain) are better at collecting a variety of cues in a short time and identifying valid cues related to target characteristics (Elstein et al., 1978; Herbig and Glöckner, 2009; Papa, 2016). We speculate that, by contrast, novice teachers’ less comprehensive and, thus, less robust reasoning might be more susceptible to premature judgments (known as premature closure in medical diagnosing; e.g., Norman et al., 2017) or otherwise biased judgments (e.g., the halo effect; Fiedler et al., 2002).

### 5.3 Limitations and future research

This study significantly advances research on teachers’ accuracy in judging student profiles. By empirically examining the differences in judgment accuracy and diagnostic reasoning between novice and experienced teachers, we employed the methodology of ENA to shed light on these differences. However, some limitations need to be addressed in future research to enhance evidence even further.

First, this study did not delve deeply into how student characteristics and profiles are manifested in students’ behavior and only took preliminary steps in this direction. The study did not address questions such as how students’ interest effectively manifests in hand-raising (see Böheim et al., 2020) or how uninterested students might obscure their low interest through adequate procedural display while in fact engaging only in mock participation (see Bloome et al., 1989; Vors et al., 2015). Subsequently, more in-depth investigations of the valid behavioral cues of different student profiles may further elucidate the relationship between experienced and novice teachers’ noticing of behavioral cues and their judgment accuracy (Herppich et al., 2018; Südkamp et al., 2018). Second, our operationalization of diagnosing consists of observing a classroom situation. In contrast, teachers’ diagnosing in real classroom situations often happens while interacting with students and engaging in intervention activities, such as instruction and classroom management. We argue that investigating diagnosing through teachers’ observation of video stimuli is advantageous in terms of standardizing the diagnostic task and setting, which is why this approach is frequently used in research on teachers’ diagnosing and professional vision (e.g., Stahnke and Blömeke, 2021). However, we acknowledge the role of research investigating diagnosing while interacting with students (in simulations, e.g., Kron et al., 2021; or in real classrooms, e.g., Südkamp et al., 2018) as well as the relation between diagnosing and intervention activities. Third, a notable limitation of our study is the lack of diversity in the authentic classroom video sequences used. We used a single video sequence, which raises questions about the generalizability of our findings. The consistency and replicability of our findings may vary if using different video sequences with different students. This highlights the potential need for further research using varied video samples to validate and solidify our current findings. Fourth, in our study, teachers were primed to include not only consistent but also inconsistent student profiles in their judgments because they were prompted to diagnose the five initially introduced profiles of student characteristics. Moreover, since the five profiles had to be assigned to five students in the video, teachers’ judgments and thus the measurements of teachers’ diagnostic accuracy and reasoning were not independent across the different students. Thus, our results might not be generalizable to teachers’ judgment accuracy regarding consistent and inconsistent student profiles in other settings. Further studies should use an unmatching number of profiles and students to be diagnosed (e.g., more or less students than profiles). In addition, research might address processes of cue comparison as well as teachers’ revisions of their judgments to better understand comparisons and references made when diagnosing multiple students. Fifth, another key limitation of our study is its limited sample size. This restricts the generalizability of our findings, as a larger, more diverse sample might reveal additional patterns or nuances, especially in teachers’ reasoning. Consequently, broader investigations are needed to confirm the robustness and applicability of our conclusions.

While acknowledging these limitations, our study underscores the importance of further investigating teachers’ judgment accuracy and reasoning when diagnosing student profiles and thereby sets the stage for future research avenues. For example, the influence of different features of student “cases” on teachers’ diagnosing might be further investigated to understand how those features contribute to making a student case difficult to reason about. Our results indicate that a higher frequency (i.e., exemplarity) a specific student profile in real classrooms might facilitate experienced teachers’ judgment accuracy because they have gained a lot of experience with students that match this frequent student profile. However, also other features of student cases and classroom situations might be worth exploring, such as the complexity of information (i.e., the amount and connectivity of information that needs to be processed), especially in terms of the salience of relevant cues (Fischer et al., 2022).

Moreover, future research might elucidate the sequence in which novice and experienced teachers employ cues for diagnostic reasoning. As inferred from our results, experienced teachers seem to leverage several surface cues, such as hand-raising, to infer deep cues, such as interest, thereby constructing more robust reasoning than novice teachers. Within the realm of educational process data mining, tools, such as the Heuristics Miner (Weijters et al., 2006), can be instrumental in discerning the most prevalent paths or sequences of cues and identifying outliers in teachers’ reasoning, while considering the chronology of cue utilization using Petri-Nets and hidden Markov models (Namaki Araghi et al., 2022). In addition, researchers could implement more complex mediator models in their analyses to explore in detail how different behavioral cues are statistically relevant in predicting or mediating teachers’ accuracy of judgment. This will require a more fine-grained and weighted coding of behavioral cues (since some cues are more or less diagnostic) and larger sample sizes.

Another potential direction for future research is leveraging the findings of this study to enhance the judgment accuracy and diagnostic reasoning of novice teachers during training sessions. For example, training sessions in study programs can discuss the outcomes of the network analysis. Future studies using data mining can contribute with further information about successful diagnostic approaches. This integration provides novice teachers with a comprehensive *blueprint* that illustrates the complex ways in which more experienced teachers use student cues in their reasoning to achieve higher accuracy in their judgments. Studies focusing on investigating perceived case difficulty conceptualized by features of student cases can further inform teacher education, for example, regarding potential sequencing strategies of different cases to facilitate novice teachers’ systematic training (see Fischer et al., 2022).

### 5.4 Conclusion

This study, anchored in the framework of teacher professional vision, delved into the diagnosing of experienced and novice teachers in terms of reasoning about student cues and judging profiles of student characteristics. By analyzing teachers’ judgment accuracy and exploring cues utilized in teachers’ written diagnostic reasoning with epistemic network analysis, our study revealed two central findings. First, experienced teachers exhibited a higher overall accuracy in judging the five student profiles. Second, experienced teachers related a higher number of cues, especially deep cues (behavioral cues that are not directly observable), in their reasoning, which was consequently more comprehensive and robust compared to the reasoning of novice teachers. This research underscores the nuanced development of professional skills, such as diagnosing, with professional experience.

## Data availability statement

The raw data supporting the conclusions of this article will be made available by the authors, without undue reservation.

## Ethics statement

Ethical review and approval was not required for the study on human participants in accordance with the local legislation and institutional requirements. The studies were conducted in accordance with the local legislation and institutional requirements. The participants provided their written informed consent to participate in this study. Written informed consent was obtained from the minor(s)' legal guardian/next of kin for the publication of any potentially identifiable images or data included in this article.

## Author contributions

CK: Conceptualization, Data curation, Formal analysis, Methodology, Writing – original draft, Writing – review & editing. EB: Formal analysis, Methodology, Writing – original draft, Writing – review & editing. TS: Conceptualization, Funding acquisition, Project administration, Resources, Writing – review & editing.

## Footnotes

^1^https://www.epistemicnetwork.org/
